# The new species *Enterobacter oryziphilus* sp. nov. and *Enterobacter oryzendophyticus* sp. nov. are key inhabitants of the endosphere of rice

**DOI:** 10.1186/1471-2180-13-164

**Published:** 2013-07-16

**Authors:** Pablo Rodrigo Hardoim, Rashid Nazir, Angela Sessitsch, Dana Elhottová, Elisa Korenblum, Leonard Simon van Overbeek, Jan Dirk van Elsas

**Affiliations:** 1 Department of Microbial Ecology, University of Groningen, Centre for Ecological and Evolutionary Studies, Nijenborgh 7, Groningen 9747AG, The Netherlands; 2 AIT Austrian Institute of Technology GmbH, Bioresources Unit, Tulln, Austria; 3 Institute of Soil Biology, Biology Centre, ASCR, v.v.i., Na Sádkách 7, České Budějovice CZ 370 05, Czech Republic; 4 Plant Research International, Droevendaalsesteeg 1, Wageningen 6708PB, The Netherlands; 5 Current address: Centre of Marine Science, University of Algarve, Faro 8005-139, Portugal

**Keywords:** Plant growth-promoting bacteria, Endophytes, Diazotrophic bacteria, Methanotrophic bacteria, Phosphate-solubilizing bacteria, Production of indole-3-acetic acid, International Rice Research Institute

## Abstract

**Background:**

Six independent Gram-negative, facultatively anaerobic, non-spore-forming, nitrogen-fixing rod-shaped isolates were obtained from the root endosphere of rice grown at the International Rice Research Institute (IRRI) and investigated in a polyphasic taxonomic study.

**Results:**

The strains produced fatty acid patterns typical for members of the family *Enterobacteriaceae*. Comparative sequence analyses of the 16S rRNA as well as *rpoB* genes allocated the strains to two well-defined groups within the genus *Enterobacter,* family *Enterobacteriaceae*. The analyses indicated *Enterobacter radicincitans*, *Enterobacter arachidis* and *Enterobacter oryzae* to be the closest related species. An RpoB (translated) protein comparison supported the placement in the genus *Enterobacter* and the relatedness of our isolates to the aforementioned species. Genomic DNA:DNA hybridization analyses and biochemical analyses provided further evidence that the novel strains belong to two new species within the genus *Enterobacter*. The two species can be differentiated from each other and from existing enteric species by acid production from L-rhamnose and D-melibiose, decarboxylation of ornithine and utilization of D-alanine, D-raffinose L-proline and L-aspartic acid, among other characteristics. Members of both species revealed capacities to colonise rice roots, including plant-growth-promoting capabilities such as an active supply of fixed nitrogen to the plant and solubilisation of inorganic phosphorus, next to traits allowing adaptation to the plant.

**Conclusions:**

Two novel proposed enterobacterial species, denominated *Enterobacter oryziphilus* sp. nov. (type strain REICA_142^T^=LMG 26429^T^=NCCB 100393^T^) and *Enterobacter oryzendophyticus* sp. nov. (type strain REICA_082^T^=LMG 26432^T^ =NCCB 100390^T^) were isolated from rice roots. Both species are capable of promoting rice growth by supplying nitrogen and phosphorus.

## Background

Plants interact with a great diversity of microorganisms, including enteric bacteria. These interactions, which are governed by the characteristics of both host plant and bacteria, result in either commensalistic, mutualistic or parasitic relationships between both partners. In rice, bacterial endophytes may provide support to the host plant when these are under stress conditions [1,2]. For instance, rice growth under conditions of low temperature, high salinity or desiccation may be favored. Moreover, endophytes can supply nitrogen to rice tissues [3]. In previous work, different bacteria, in particular belonging to the enterics, have been isolated from rice seeds [4,5], roots [3,6] and stems [7]. For example, *Enterobacter cloacae* subsp*. dissolvens*, previously described as *Erwinia dissolvens*, was first isolated from diseased corn [8], whereas it was also found in the endosphere of rice plants without causing apparent harm to the host plant [9]. *Enterobacter cancerogenus* NCPPB 2176^T^, *E. nimipressuralis* ATCC 9912^T^ and *E. pyrinus* ATCC 49851^T^ were isolated from symptomatic necrosis sites, respectively from poplar, elm and pear trees [8,10,11]. These organisms are therefore known as phytopathogens. On the other hand, organisms such as *E. radicincitans* D5/23^T^, *E. arachidis* Ah-143^T^, *E. oryzae* Ola-51^T^ and *Enterobacter* sp. CBMB30, which have been isolated from respectively the phyllosphere of wheat, the rhizosphere of groundnut and the endosphere of rice species (i.e. *Oryza latifolia* and *O. sativa*), can improve the fitness of their host plants and are therefore known as plant-growth-promoting bacteria (PGPB; [3,12,13]).

In a recent study, we assessed the bacterial communities that occur within roots of rice plants by both cultivation-independent (i.e. more than 500 clones containing the 16S rRNA gene were sequenced) and cultivation-dependent approaches [14]. From the directly-obtained clone library, ca. 30% of the sequences were assigned to one unique operational taxonomic unit (OTU), defined at 99% sequence similarity as a member of the genus *Enterobacter*. In addition, we obtained a high number of bacterial isolates (222) from the same samples, by serial dilution on R2A agar. After screening these isolates to assess the number of different genotypes via BOX-A1R PCR, 84 distinct fingerprinting patterns were observed across all, using an 80% similarity cut-off level [14]. Preliminary analysis of the 16S rRNA genes of each of these groups revealed a suite of six independent (non-clonal) strains that were closely related to the most abundantly retrieved OTU from the clone library. This clearly demonstrated the predominance of *Enterobacter-*related types in the rice root bacterial community and indicated their potential functional importance. The 16S rRNA sequences also matched a sequence obtained from an *Enterobacter* sp. (denoted CBMB30), a rice endophytic bacterium isolated in South Korea that was reported to have plant-growth-promoting properties [15].

In the current study, the six strains, divided into two related groups of three strains each, are further characterized. On the basis of the collective results obtained, we propose that they constitute two new species, which we denominate *Enterobacter oryziphilus* sp. nov. (strains REICA_084, REICA_142^T^ and REICA_191) and *Enterobacter oryzendophyticus* sp. nov. (strains REICA_032, REICA_082^T^ and REICA_211).

## Results and discussion

### Presumptive identification of strains

**Figure 1:**
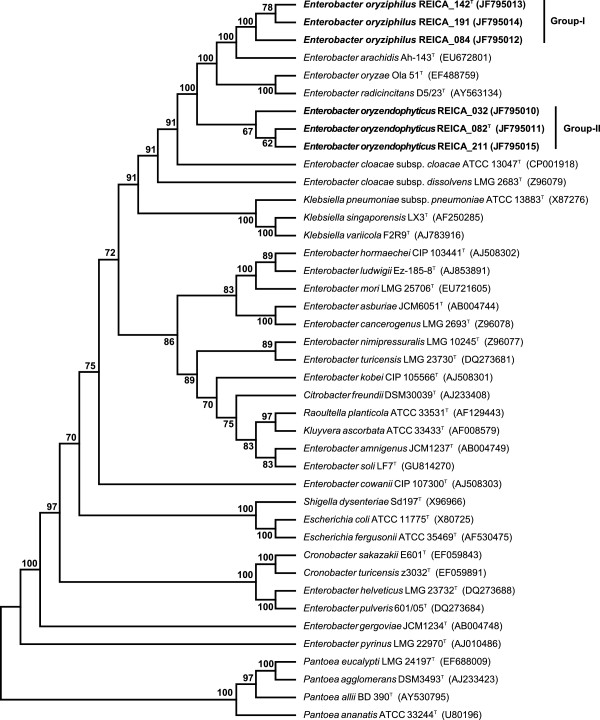
**Maximum parsimony (MP) strict consensus tree based on the 16S rRNA gene sequences of selected *Enterobacteriaceae*.** Tree was constructed using CNI with a search level of 3, and initial trees by random addition (100 reps). The consensus tree inferred from 58 optimal trees is shown. Branches corresponding to partitions reproduced in less than 50% trees are collapsed. The percentage of parsimonious trees in which the associated taxa cluster together in the bootstrap test (1000 replications) are shown next to the branches. The analyses encompassed 41 nucleotide sequences. All positions containing gaps and missing data were eliminated. There was a total of 1125 positions in the final dataset. Evolutionary analyses were conducted in MEGA5.

Six isolates, obtained from different rice root samples, were grouped, by preliminary analyses, into two groups of three strains each, which both resembled, by comparison of their partial 16S rRNA gene sequences, the dominant clones in a directly obtained clone library [14]. Analyses of the full 16S rRNA gene sequences of all isolates then revealed hits, at high levels of homology, with sequences belonging to members of the genus *Enterobacter*, including the type strains of several different species. Figure 1 gives a depiction of a maximum parsimony (MP) based phylogenetic tree, which used 1125 unambiguously aligned positions, 90 of which are informative under the parsimony criterion. The tree was constructed on the basis of a comparison of the six new isolates with a range of related (mostly *Enterobacter*) sequences. The topology of the tree was strongly supported by bootstrap analyses (Figure 1). Phylogenetic inference on the basis of maximum likelihood was also performed and yielded the same result as that obtained with the MP based trees (Additional file 1: Figure S1). Thus, on the basis of the 16S rRNA gene sequences, strains REICA_142, REICA_084 and REICA_191 were identical and formed a separate branch in the tree that indicated a novel phylogenetic group (I). Moreover, the sequences of the remaining three novel strains, i.e. REICA_082, REICA_032 and REICA_211, were virtually identical to each other (99.9% sequence similarity) and formed another separate branch (denoted II) in the tree. Again, this branch was strongly supported by bootstrap analyses (Figure 1). This 16S rRNA gene based analysis provided preliminary evidence for the contention that both groups of strains, I and II, may form the core of two novel rice-interactive *Enterobacter* species.

One strain of group-I, i.e. REICA_142, was then selected as the putative type strain of a novel taxon, denoted REICA_142^T^. It revealed closest relatedness, at the level of the 16S rRNA gene sequence, to *E. arachidis* Ah-143^T^ (99.3% sequence similarity), *E. oryzae* Ola-51^T^ (98.8%), *E. radicincitans* D5/23^T^ (98.5%) and *E. cloacae* subsp. *cloacae* ATCC 13047^T^ (98.0% sequence similarity). Moreover, strain REICA_082 of group-II was taken as the putative type strain of another novel taxon (i.e. REICA_082^T^). This taxon was most closely related (16S rRNA gene) to *E. cloacae* subsp. *cloacae* ATCC 13047^T^ (99.3% sequence similarity), *E. cloacae* subsp. *dissolvens* ATCC 23373^T^ (99.0%), *E. arachidis* Ah-143^T^ (98.9%) and *E. oryzae* Ola-51^T^ (98.7%).

**Figure 2:**
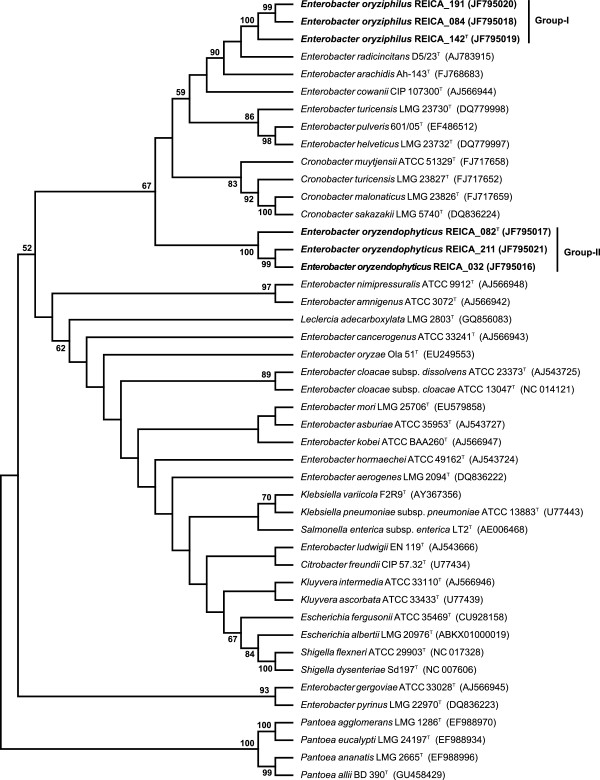
**Maximum parsimony (MP) consensus tree based on the *rpoB *gene sequence of selected *Enterobacteriaceae*.** Tree wasconstructed using CNI with a search level of 3, and initial trees by random addition (100 reps). The consensus tree inferred from 5600 most parsimonious trees is shown. Branches corresponding to partitions reproduced in less than 50% of the trees are collapsed. The percentage of parsimonious trees in which the associated taxa cluster together in the bootstrap test (1000 replications) are shown next to the branches. The analyses involved 45 sequences. All positions containing gaps and missing data were eliminated. There was a total of 495 positions in the final dataset, 136 of which are informative under the parsimony criterion. Evolutionary analyses were conducted in MEGA5.

However, classification on the basis of a single phylogenetic marker, in particular the 16S rRNA gene, has known caveats for species within the genus *Enterobacter*. The genus itself is poorly definable. To overcome such taxonomic difficulties, it has been proposed that a second phylogenetic marker, i.e. *rpoB*, should be used for the identification of species within the *Enterobacteriaceae,* including *Enterobacter*[16]. The *rpoB* gene encodes the *β*-subunit of RNA polymerase and is part of the core genome of *Enterobacter*. This gene has higher discriminatory power than the 16S rRNA gene and has been recommended for use in a more robust allocation of new species [16]. Moreover, similarities in sequences of the *rpoB* gene have been found to be highly correlated with those of the G+C% of bacterial genomes, as well as with DNA:DNA hybridization values [17]. Thus, *rpoB* has become an important proxy in studies aiming for the discrimination of closely-related strains and species. A comparison of the *rpoB* gene sequences of all six strains and their closest neighbours (Figure 2) revealed that all novel sequences were less than 98% similar to any of the described sequences. Given the fact that the 98% level of *rpoB* gene sequence similarity represents the proposed cut-off level for the definition of species within the family *Enterobacteriaceae*[16], this yielded a second piece of evidence for the contention that the two groups of new strains constitute novel species within the *Enterobacteriaceae*. Figure 2 further showed that the *rpoB* sequences of strains of group-I (REICA_142^T^, REICA_084 and REICA_191) were identical to each other, grouping distantly with a cluster containing sequences of *E. radicincitans* D5/23^T^ (97.5% similarity), *E. arachidis* Ah-143^T^ (96.6%) and *E. cowanii* CIP 107300^T^ (92.8%). The *rpoB* gene sequences of the group-II strains were also virtually identical, with those of strains REICA_032 and REICA_211 being the same and 99.8% similar to that of REICA_082^T^. As these sequences were quite divergent from those of any other group (as well as from the first group), a separate cluster was defined in the tree (Figure 2). The sequence of the proposed group-II type strain REICA_082^T^ was most closely related to that of *E. radicincitans* D5/23^T^ (92.4% sequence similarity), *E. arachidis* Ah-143^T^ (92.0%) and strain REICA_142^T^ (91.9%). Phylogenetic inference on the basis of maximum likelihood corroborates the results obtained with the MP based trees (Additional file 2: Figure S2). Additionally, the *rpoB* gene based analyses were supported by those of the predicted proteins; in these nucleotide sequence based analyses, the strains of groups I and II again clustered tightly together within a main cluster encompassing a range of *Enterobacter* (next to *Cronobacter*) strains including the same close relatives as above (data not shown).

In the light of the results obtained with the two-pronged approach based on the 16S rRNA and *rpoB* gene sequence analyses, we posit that the six novel rice endophytic strains represent two different species within the genus *Enterobacter*. However, this genus is currently undergoing a re-examination. For instance, a novel genus termed *Cronobacter,* has been recently coined, as a split-off of particular species/strains belonging to the group. We found that the *rpoB* sequences of the two type strains of our novel proposed species groups, REICA_142^T^ and REICA_082^T^, were quite distantly related to those of the type species *E. cloacae* subsp. *cloacae* ATCC 13047^T^ (89.3 and 90.5% sequence similarities, respectively) and *Cronobacter sakazaki* LMG 5740^T^ (90.5 and 90.1%, respectively). These values are actually well below the reasonable limit of 6% sequence dissimilarity, which has been proposed to differentiate genera within the *Enterobacteriaceae*[18]. In the future, these might be focal points for the definition of novel genera. It is interesting that both the 16S rRNA gene and the *rpoB* gene sequence based phylogenetic analyses revealed the existence of robust clades (supported by MP bootstrap values of 100%, Figures 1 and 2), in which our novel group-I strains (REICA_142^T^, REICA_084 and REICA_191) were most related to the *Enterobacter* type strains *E. radicincitans* D5/23^T^ and *E. arachidis* Ah-143^T^. It is important to remark that the latter strains have previously been shown to improve plant growth by increasing the root length, as well as the (dry) mass, of several host plants [19]. Therefore, an understanding of the ecology of our novel strains will add to a growing body of knowledge on the species diversity of *Enterobacter* types in rice roots. Ecological behaviour is locked in into taxonomy in particular with respect to those traits that define phenotype. Given the fact that a sound species definition depends on a combination of techniques, including an analysis of genomic DNA relatedness, we determined the DNA:DNA homologies among a selection of our novel and closely-related strains.

### Genomic DNA:DNA hybridization analyses confirm the existence of two novel *Enterobacter* species

Pairwise genomic DNA hybridization tests (Table 1) were performed across a selection of four strains of the two newly defined species (two each, including the two proposed type strains) and the closest relatives *E. arachidis* LMG 26131^T^, *E radicincitans* LMG 23767^T^, *E. cowanii* LMG 23569^T^ and *E. oryzae* LMG 24251^T^ (see above). First, these analyses revealed that the group-I strains REICA_142^T^ and REICA_191 and the group-II ones REICA_082^T^ and REICA_032 had high within-group DNA:DNA relatedness (93 and 89%, respectively), whereas the putative type strains REICA_142^T^ (group-I) and REICA_082^T^ (group-II) had low (38% ±10) DNA:DNA relatedness between them. These results suggested a taxonomic tightness within the two groups, versus a low relatedness between them. Taking the 70% similarity criterion as the basis for the separation or junction of species, the data provided sound evidence for the contention that the two groups constitute disparate species.

**Table 1:** **DNA:DNA relatedness percentages between representatives of two novel *Enterobacter * species and closely-related species**

	**1**	**2**	**3**	**4**	**5**	**6**	**7**	**8**
**1**	100							
**2**	89(4)	100						
**3**	33(16)	38(10)	100					
**4**	31(17)	33(10)	93(6)	100				
**5**	35(2)	33(9)	35(17)	31(7)	100			
**6**	32(10)	35(2)	59(7)	58(3)	33(2)	100		
**7**	39(9)	41(3)	/	61(9)	43(8)	79(6)	100	
**8**	33(8)	31(1)	63(8)	60(14)	33(21)	66(17)	71(2)	100

The data are based on means of at least 4 hybridizations. The values given between brackets are the differences between the reciprocal values. Taxa: 1, *Enterobacter oryzendophyticus* REICA_032; 2, *Enterobacter oryzendophyticus* REICA_082^T^; 3, *Enterobacter oryziphilus* REICA_142^T^; 4, *Enterobacter oryziphilus* REICA_191; 5, *Enterobacter cowanii* LMG 23569^T^; 6, *Enterobacter radicincitans* LMG 23767^T^; 7, *Enterobacter oryzae* LMG 24251^T^; 8, *Enterobacter arachidis* LMG 26131^T^.

Furthermore, group-I type strain REICA_142^T^ DNA showed only about 35-60% relatedness with the DNA of the closest relatives *E. arachidis* LMG 26131^T^ (63% ±8), *E radicincitans* LMG 23767^T^ (59% ±7) and *E. cowanii* LMG 23569^T^ (35% ±17). This finding is consistent with the contention that the group-I strains indeed form a separate species, within the genus *Enterobacter*. Similarly, strain REICA_082^T^ genomic DNA revealed relatedness values that were significantly below the 70% cut-off value with that of the closest-related strains *E. oryzae* LMG 24251^T^ (41% ±3), *E. radicincitans* LMG 23767^T^ (35% ±2), *E. cowanii* LMG 23569^T^ (33% ±9) and *E. arachidis* LMG 26131^T^ (31% ±1) (Table 1). Again, this finding supports our contention that also the group-II strains form a separate species within the genus *Enterobacter*.

It was interesting to note that the DNA-DNA relatedness values between *E. radicincitans* LMG 23767^T^ and *E. oryzae* LMG 24251^T^ (79% ±6) and between *E. radicincitans* LMG 23767^T^ and *E. arachidis* LMG 26131^T^ (66% ±17), in our experiments, were much higher than those reported by the original authors [3]. Support for the robustness of our data is provided by the phylogenetic relationships revealed by the *rpoB* gene sequences, where *E. radicincitans* D5/23^T^ and *E. arachidis* Ah-143^T^ were 98.9% similar. These data were further consistent with the cellular fatty acid profile data (see below), which were indistinguishable at strain level.

The overall genomic DNA G+C content was determined according to the HPLC method [20] using the DNA prepared for the DNA:DNA hybridization analyses. The values (means of three independent analyses of the same DNA sample) for the selected group-II strains REICA_032 and REICA_082^T^ and group-I strains REICA_142^T^ and REICA_191 were 52.7, 52.9, and 52.1 and 51.7 mol%, respectively. These values are within the lower range of the DNA mol% *G* + *C*, i.e. 52–60 %, of all members of the genus *Enterobacter*[21].

### Fatty acid methyl ester (FAME) profiling confirms the allocation of group-I and group-II strains into two novel *Enterobacteriaceae* species

The FAME profiles obtained by using the MIDI system from all six novel strains were similar to those of several type strains of the aforementioned *Enterobacter* species. However, there were differences in the relative proportions of particular fatty acids. First, based on the data, the six strains could again be separated into the exact same two groups, denoted I (REICA_142^T^, REICA_084 and REICA_191) and II (REICA_082^T^, REICA_032 and REICA_211). Expectedly, the putative type strains of the two groups shared some commonalities, as the predominant cellular fatty acids of group-I strain REICA_142^T^ and group-II strain REICA_082^T^ were C_16:0_ (34.3 and 32.7%, respectively), summed feature 8 (C_18:1_*ω*7*c* and/or C_18:1_*ω*6*c* with 19.2 and 27.6%), summed feature 3 (C_16:1_*ω*7*c* and/or C_16:1_*ω*6*c* with 20.7 and 26.4%) and C_17:0_ cyclo (14.2 and 4.9%). Moreover, fatty acids C_14:0_ and C_12:0_ were also found (Additional file 3: Table S1). Although it is known that the (ITSA – instant trypticase soy agar) library of the MIDI (microbial identification, Inc) system is incomplete and provides somewhat biased results, a comparison with this database resulted in the remote affiliation of group-I strain REICA_142^T^ with *Salmonella enterica* subsp. *enterica* and/or *Serratia marcescens* (similarity index > 0.6) and that of group-II strain REICA_082^T^ with *Klebsiella mobilis*, *Escherichia coli*, *Escherichia fergusonii* and *K. pneumoniae* subsp. *pneumoniae* (similarity index > 0.55). However, environmental enteric strains are underrepresented in this database and an update is needed to allow any robust taxonomic assignment of environmental strains.

A dendrogram constructed on the basis of the above data indicated that the selected group-I and group-II representatives cluster within the *Enterobacteriaceae*, but not within any known species (Additional file 4: Figure S3). Thus, group-I strain REICA_142^T^ was related to *Enterobacter cloacae* subsp. *cloacae* subgroup C, whereas it also resembled *Serratia marcescens* subgroup C and *Klebsiella oxytoca* subgroup B. Moreover, group-II strain REICA_082^T^ was related to *E. coli* subgroups C and E, *E. fergusonii* subgroup A, *K. mobilis* and *Salmonella enterica* subsp. *houtenae* (Additional file 4: Figure S3). The cellular fatty acid profile of *E. arachidis* Ah-143^T^ was highly similar to that of *E. radicincitans* D5/23^T^, with a Euclidian distance below 2.5 (Additional file 4: Figure S3). Both strains formed a distinct cluster related to *Leclercia adecarboxylata* subgroup A, *Citrobacter freundii*, *K. oxytoca* subgroup D and *S. marcescens* subgroup D.

### Novel species descriptions

Cells of all novel strains, i.e. REICA_142^T^, REICA_084, REICA_191 (group-I) and REICA_082^T^, REICA_032 and REICA_211 (group-II), were facultatively anaerobic, Gram-negative, motile and straight rod-shaped (0.8-1.0 × 1.8-3.0 μm). After 24 h incubation at 37°C on TSA, the colonies were flat, translucent, regularly-shaped and beige-pigmented. After an extended period of incubation, colonies of group-II strain REICA_082^T^ showed filiform margins, whereas those of the other strains did not show this phenomenon. All strains grew at temperatures between 15 and 42°C and in the presence of up to 5% NaCl. The putative type strains REICA_142^T^ (group-I) and REICA_082^T^ (group-II) were resistant to ampicillin (25 μg), colistin sulphate (100 μg), kanamycin (30 μg), nitrofurantoin (50 μg) and streptomycin (25 μg). However, they were sensitive to rifampicin and gentamicin (25 μg ml^-1^), chloramphenicol (50 μg) and tetracycline (100 μg). Strain REICA_082^T^ was resistant to nalidixic acid (30 μg). On the other hand, strain REICA_142^T^ was not.

All group-I and group-II strains were catalase-positive and oxidase-negative and revealed physiological and biochemical characteristics similar to those of other strains of the genus *Enterobacter*[21,22]. They could be differentiated from species in closely-related genera, i.e. *Klebsiella, Escherichia* and *Salmonella,* as follows. The novel (group I and II) *Enterobacter* species were positive for arginine dihydrolase, showed motility and were negative for the utilization of quinic acid. In contrast, *Klebsiella* species are non-motile (except for *Klebsiella mobilis*), are arginine-negative and are capable to utilize quinic acid. The novel (group I and II) species produced acetoin (Voges-Proskauer test) but not indole. In contrast, *Escherichia* species are acetoin-negative but produce indole. Interestingly, indole production has also been observed in *Cronobacter* species, and hence the two new species were differentiated from *Cronobacter*. The group-I and group-II strains were all negative for the production of hydrogen sulphide, where, in contrast, species of *Salmonella* are positive.

Notwithstanding the limitations of the API 20E biochemical test database, it was applied for all strains of group I and II, next to the closely-related comparator strains (Table 2). On the basis of the API 20E system, the six strains fell precisely into the two groups (I and II), as delineated in the foregoing. These were differentiated by the following characteristics: group-I strains REICA_142^T^, REICA_084 and REICA_191 were positive for D-alanine, L-alanylglycine, L-aspartic acid and L-glutamic acid. At least one of these strains was also positive for the utilization of cis-aconitic acid and L-histidine. On the other hand, the group-II strains REICA_082^T^, REICA_032 and REICA_211 could utilize the following substrates as sole carbon sources: D-raffinose, malonic acid, *β*-hydroxybutyric acid, Tween 40, L-proline, inosine and thymidine. At least one of these strains was positive for the utilization of D-melibiose, α-cyclodextrin, acetic acid, formic acid and glycogen. The discriminatory properties of the two novel species and closely related species are given in Table 2.

**Table 2:** **Key reactions for biochemical differentiation of selected *Enterobacter *species**

**Characteristic**^**a**^	**1**	**2**	**3**	**4**	**5**	**6**	**7**	**8**
Voges-Proskauer test (37°C)^b^	+	+	+	-	-	-	+	+
Methyl red test	+	+	+	+	+	+	-	-
Cell morphology	SR	SR	R	CR	CR	CR	R	R
Ornithine decarboxylase^b^	-	+	-	-	-	-	+	+
Malonate decarboxylase (48 h)	+	+	+	-	+	+	-	+
Arginine dehydrolase^b^	+	+	+	-	-	-	+	+
Esculin hydrolysis	+	+	+	-	-	+	-	+
Citrate^b^	+	+	+	+	-	-	+	+
Gluconate dehydrogenase	+	+	+	-	+	+	-	ND
Carbon source utilization^c^								
Sucrose	+	+	+	-	-	+	+	+
D-melibiose	-	V	-	+	+	+	+	+
Adonitol	+	+	-	-	-	-	-	-
D-sorbitol	+	+	+	-	-	-	+	+
L-fucose	+	V	ND	-	-	-	-	-
L-aspartic acid	+	-	+	+	+	+	ND	ND
m-inositol	+	+	-	-	-	-	+	+
D-arabitol	+	+	-	-	-	+	-	-
D-raffinose	-	+	-	-	-	+	+	+

^a^For all strains analysed, the following tests were positive: catalase, *β*-galactosidase (ONPG) and motility and negative for: oxidase, lysine decarboxylase, urease, indole and H_2_S production.

^b^Test results of both the API-20E system and conventional test methods.

^c^The carbon source utilization tests were determined by using Biolog GN2 microplates.

*Species: 1, *Enterobacter oryziphilus* sp. nov. (n=3); 2, *Enterobacter oryzendophyticus* sp. nov. (n=3); 3, *Enterobacter radicincitans* D5/23^T^; 4, *Enterobacter turicensis* 508/05^T^; 5, *Enterobacter helveticus* 513/05^T^; 6, *Enterobacter pulveris* 601/05^T^. 7, *Enterobacter cloacae* subsp. *cloacae;* data from [23-25]; 8, *Enterobacter cloacae* subsp. *dissolvens*, data from [8,26]. The percentage of strains giving a positive result is scored as: -, 0–20%; V, 20–80%; +, 80–100%; ND, no data available; cell morphology: R, rods; CR, coccoid rods; SR, straight rods.

The putative type strains REICA_142^T^ and REICA_082^T^ were then compared to the API20E database. The database revealed as closest relative for the group-I type strain REICA_142^T^*Enterobacter asburiae* (only 29% identity) and for group-II type strain REICA_082^T^*E. cloacae* (95% identity), respectively. The two strains differed mainly in the ornithine decarboxylase test and in the production of acid from L-rhamnose and D-melibiose (all reactions positive in strain REICA_082^T^). However, this database is as limited as the MIDI one discussed earlier, and environmental strains are needed to make it suitable for environmental work.

### Plant-beneficial and adaptive traits

Finally, we evaluated the capacities of strains REICA_142^T^ (group-I) and REICA_082^T^ (group-II) to modulate rice plant growth and to colonize rice host plants from soil. Group-II strain REICA_082^T^ produced indole acetic acid (IAA; 4.12 μg ml^-1^; ±0.68) from L-tryptophan, whereas group-I strain REICA_142^T^ did not. Both strains revealed the production of acetoin, 2-ketogluconate via gluconate dehydrogenase and siderophores (after 24 h at 30°C), and the solubilisation of phosphate via acidification but not alkalinisation. 2-ketogluconate is the salt compound of the organic acid 2-ketogluconic acid. This organic acid is produced by phosphate-solubilizing bacteria (PSB) and is known to be involved in the solubilisation of inorganic phosphates [27]. Cellulase activity, as well as growth on “copiotrophic” and “oligotrophic” media, were observed for both strains, but no amylase and protease activities were registered. Strain REICA_082^T^ showed growth on M9 salt agar amended with methanol, but strain REICA_142^T^ did not. Supporting evidence for the transformation of methanol was provided by the finding that the gene encoding the alpha subunit of methanol dehydrogenase could be amplified from the REICA_082^T^ genome (550 bp). To the best of our knowledge, only two other *Enterobacter* strains (Ah-143^T^ and CBMB30) have previously been shown to be able to use methanol as the sole carbon and energy source [13,15]. In semi-solid Rennie medium (0.2% agar), strains REICA_142^T^ and REICA_082^T^ reduced, respectively, 3.66% (±0.02) and 0.24% (±0.0002) of acetylene to ethylene during 24 h of incubation at 37°C, indicating their nitrogen fixing capacity. As a control, bacterial cells that had been inactivated after boiling the liquid culture for 10 min did not show acetylene reduction. Moreover, the presence of the gene encoding nitrogen reductase could be shown in both organisms using PCR (amplicons of ca. 350 bp). These results show that both bacteria are diazotrophic and may be capable of establishing endophytic associations with rice and growth in plant tissue, most likely without causing any harm to the host.

Therefore, the rifampicin-resistant derivative of strain REICA_142, denoted REICA_142^TR^, was tested for colonization and growth *in planta* in a colonization experiment with young rice seedlings to which the strain was introduced. All replicate rice seedlings growing in gamma-sterlized as well as natural soil showed invasion by strain REICA_142^TR^. Plants growing in strain REICA_142^TR^ treated pre-sterilized soil revealed populations of 6.3±0.6 *log* CFU g^-1^ fresh root tissue and 4.1±0.4 *log* CFU g^-1^ fresh shoot tissue, whereas plants from non-presterilized soil treated with the same strain revealed lower numbers of cells, i.e. 4.6±0.4 *log* CFU g^-1^fresh root tissue and 3.6±0.3 *log* CFU g^-1^ fresh shoot tissue. No bacterial growth was observed on plates that received homogenates from rice plants growing in uninoculated soils (all dilutions), leading to the conclusion that their numbers were below 2.0 *log* CFU g^-1^ fresh weight. Under the experimental conditions used, no significant differences in plant fresh weight (g) were noticed between inoculated and control plants. In sterile soil, the fresh weight of rice seedlings growing in the presence of strain REICA_142^TR^ was 0.83 g (±0.44), while plants growing without this strain weighed 0.82 g (±0.26). However, the introduction of strain REICA_142^TR^ apparently did alter plant physiology, albeit below statistical significance (P > 0.05). Thus increases of 40% and decreases of around 9% in the root and shoot fresh weights, respectively, were noted. It is interesting to note that the beneficial effect of plant-growth-promoting bacteria is often associated with the inoculant population density. For instance, a significant increase in the growth of young *Brassica oleracea* plants was only observed at high inoculum level of *E. radicincitans* D5/23T (about 9 *log* CFU per plant), but not at a lower level, i.e. 8 *log* CFU per plant [19]. Rice plants growing in non-sterile soil revealed reduced fresh weights, i.e. 0.31 g (±0.07) for uninoculated plants and 0.30 g (±0.08) for inoculated ones. The initial microbiota in the unsterilized soil thus appeared to impair the growth of rice plants, when compared to sterilized soil. In a recent review, Reinhold-Hurek and Hurek [28] addressed the recalcitrance of bacterial endophytes to cultivation. Many abundant endophytes that are active *in planta* are still uncultivable. In addition, the already cultivated ones are often scarcely culturable *in planta*. We here provide evidence for the existence of two novel culturable *Enterobacter* species in the rice endosphere. The group-I strain REICA_142^TR^ was remarkable, as it is easily cultivated *in vitro* as well as *in planta*. Besides*,* this strain was related to a dominant gene sequence found in the library representing rice root endophytes [14].

## Conclusions

### Arguments for the definition of two novel Enterobacter species

On the basis of the foregoing data and arguments for the importance and relevance of rice-associated *Enterobacter* species, we propose that the group-I and group-II strains are classed into two novel species that should - considering the genus is intact at this point in time - be placed inside the genus *Enterobacter*. First, both groups are internally very homogeneous, and, by all criteria used, they class as solid taxonomic units. Secondly, on the basis of (1) the 16S rRNA gene sequence similarity, (2) the *rpo*B gene sequence similarity and (3) the DNA:DNA hybridization data, we clearly discern the appearance of two novel groups (radiations) within the genus *Enterobacter*. These two strain groups are thus proposed to form two novel species, denoted *Enterobacter oryziphilus* and *Enterobacter oryzendophyticus*. Both groups are likely to have their preferred niche in association with rice plants. They may play key roles in the rice endosphere, providing an ecologically-based justification for their definition. The descriptions of the two species are given below.

#### Description of *Enterobacter oryziphilus* sp. nov

*Enterobacter oryziphilus*: o.ry.zi´phi.lus. L. nom. n. *oryza*, rice; *philus* (from Gr. masc. adj. *philos*), friend, loving; N.L. masc. adj. oryziphilus, rice-loving.

Cells are Gram-negative, motile, straight rods (0.9-1.0 μm wide by 1.8-2.9 μm long) and occur singly or in pairs. Mesophilic, chemoorganotrophic and aerobic to facultatively anaerobic. Colonies on TSA medium are beige pigmented, 2–3 mm in diameter and convex after 24 h at 37°C. Growth occurs at 15-42°C (optimum 28-37°C). NaCl inhibits growth at concentrations above 5%. Growth was detected on C and O media. Cytochrome oxidase negative and catalase positive. The type strain is resistant to ampicillin (25 μg), colistin sulphate (100 μg), kanamycin (30 μg), nitrofurantoin (50 μg) and streptomycin (25 μg); however, it is sensitive to rifampicin (25 μg ml^-1^) and gentamicin (25 μg ml^-1^), nalidixic acid (30 μg), chloramphenicol (50 μg) and tetracycline (100 μg). Showed a positive reaction for Voges–Proskauer, arginine dihydrolase, gluconate dehydrogenase, malonate decarboxylase, esculin hydrolysis, ONPG (*ortho*-nitrophenyl-β-galactoside) hydrolysis, methyl red test, reduction of nitrate and alkaline reaction occurs in Simmons citrate agar; revealed to be negative for urease, gelatin hydrolysis, H_2_S production, indole production, tryptophan deaminase, lysine decarboxylase and ornithine decarboxylase. Acid is produced from the following compounds: D-glucose, D-mannitol, D-sorbitol, D-sucrose, L-arabinose and amygdalin. No acid production is observed from D-melibiose, L-rhamnose and inositol. Acetylene reduction, production of acetoin and siderophore, phosphate solubilisation and cellulase are positive, whereas amylase, protease and production of IAA are negative. Positive for utilization of adonitol, L-arabinose, D-arabitol, D-cellobiose, D-fructose, L-fucose, D-galactose, gentiobiose, *α*-D-glucose, m-inositol, α-D-lactose, lactulose, maltose, D-mannitol, D-mannose, *β*-methyl-D-glucoside, D-psicose, L-rhamnose, D-sorbitol, sucrose, D-trehalose, turanose, xylitol, pyruvic acid methyl ester, succinic acid mono-methyl-ester, citric acid, D-galacturonic acid, D-gluconic acid, D-glucosaminic acid, D-glucuronic acid, D,L-lactic acid, D-saccharic acid, succinic acid, bromosuccinic acid, glucuronamide, L-alaninamide, D-alanine, L-alanine, L-alanyl-glycine, L-asparagine, L-aspartic acid, L-glutamic acid, L-serine, glycerol, D,L-*α*-glycerol phosphate, *α*-D-glucose-1-phosphate, D-glucose-6-phosphate, dextrin, Tween 80, N-acetyl-D-galactosamine and *N*-acetyl-D-glucosamine. The following compounds are not utilized as sole carbon source: *i*-erythritol, D-melibiose, D-raffinose, acetic acid, formic acid, cis-aconitic acid, D-galactonic acid lactone, *α*-hydroxybutyric acid, *β*-hydroxybutyric acid, *γ*-hydroxybutyric acid, *p*-hydroxy phenylacetic acid, itaconic acid, *α*-keto butyric acid, *α*-keto glutaric acid, *α*-keto valeric acid, malonic acid, propionic acid, quinic acid, sebacic acid, succinamic acid, glycyl-L-aspartic acid, glycyl-L-glutamic acid, hydroxy-L-proline, L-histidine, L-leucine, L-ornithine, L-phenylalanine, L-proline, L-pyroglutamic acid, D-serine, L-threonine, D,L-carnitine, *γ*-amino butyric acid, urocanic acid, inosine, uridine, thymidine, phenylethylamine, putrescine, 2-aminoethanol, 2,3-butanediol, *α*-cyclodextrin, glycogen and Tween 40. The *nifH* gene for nitrogenase reductase was detected in the genomic DNA, but not the *mxaF* gene for methanol dehydrogenase for strains REICA_142^T^, REICA_084 and REICA_191. The genomic DNA G+C contents of strains REICA_142^T^ and REICA_191 are 52.1 and 51.7 mol%, respectively. The 16S rRNA and *rpoB* gene sequences are deposited under the accession numbers [GenBank:JF795013, JF795019] for REICA_142^T^, respectively.

The type strain, REICA_142^T^ (= LMG 26429 =NCCB 100393^T^), was isolated from internal root tissues of rice (*Oryza sativa* L.) cultivar APO. The samples were collected at flowering stage from an experimental paddy field at the IRRI, Philippines.

#### Description of *Enterobacter oryzendophyticus* sp. nov.

*Enterobacter oryzendophyticus*: o.ry.za.*en.do.phy´ti.cus.* L. n. *oryza*, rice; Gr. pref. *endo*-, within; Gr. neutr. n. *phyton*, plant; L. masc. suff. -*icus*, suffix used with the sense of pertaining to; N.L. masc. adj. oryzendophyticus , within rice plant, pertaining to the original isolation from rice tissues).

Cells are Gram-negative, motile, straight rods (0.8-1.0 μm wide by 1.8-3.0 μm long) and occur singly or in pairs. Mesophilic, methylotrophic, chemoorganotrophic and aerobic to facultatively anaerobic. Colonies on TSA medium are beige pigmented, 1–1.5 mm in diameter and convex after 24 h at 37°C. Growth occurs at 15-42°C (optimum 28-37°C). NaCl inhibits growth at concentrations above 5%. Growth was detected on C and O media and on M9 salt amended with 1% (v/v) methanol as sole carbon source. Cytochrome oxidase negative and catalase positive. The type strain is resistant to ampicillin and streptomycin (25 μg), kanamycin and nalidixic acid (30 μg), nitrofurantoin (50 μg) and colistin sulphate (100 μg); however, sensitive to rifampicin and gentamicin (25 μg ml^-1^), chloramphenicol (50 μg) and tetracycline (100 μg). Showed a positive reaction for Voges–Proskauer, arginine dihydrolase, gluconate dehydrogenase, malonate and ornithine decarboxylase, esculin hydrolysis, ONPG hydrolysis, methyl red test, reduction of nitrate and alkaline reaction occurs in Simmons citrate agar; negative for urease, gelatin hydrolysis, H_2_S production, indole production, tryptophan deaminase and lysine decarboxylase. Acid is produced from the following compounds: D-glucose, D-mannitol, D-sorbitol, D-sucrose, D-melibiose, L-rhamnose, L-arabinose and amygdalin. No acid production is observed from inositol. Acetylene reduction, phosphate solubilization, cellulase and production of IAA, acetoin and siderophore were positive, while amylase and protease were negative. Positive for utilization of adonitol, L-arabinose, D-arabitol, D-cellobiose, D-fructose, L-fucose, D-galactose, gentiobiose, *α*-D-glucose, m-inositol, α-D-lactose, lactulose, maltose, D-mannitol, D-mannose, D-melibiose, *β*-methyl-D-glucoside, D-psicose, D-raffinose, L-rhamnose, D-sorbitol, sucrose, D-trehalose, turanose, xylitol, pyruvic acid methyl ester, succinic acid mono-methyl-ester, acetic acid, bromosuccinic acid, citric acid, formic acid, D-galacturonic acid, D-gluconic acid, D-glucosaminic acid, D-glucuronic acid, *β*-hydroxybutyric acid, D,L-lactic acid, malonic acid, D-saccharic acid, succinic acid, glucuronamide, L-alaninamide, L-alanine, L-asparagine, L-histidine, L-proline, L-serine, inosine, thymidine, glycerol, D,L-*α*-glycerol phosphate, *α*-D-glucose-1-phosphate, D-glucose-6-phosphate, dextrin, *α*-cyclodextrin, glycogen, Tween 40, Tween 80, N-acetyl-D-galactosamine and *N*-acetyl-D-glucosamine. The following compounds were not utilized as sole carbon source: *i*-erythritol, *α*-hydroxybutyric acid, *α*-keto butyric acid, *α*-keto glutaric acid, *α*-keto valeric acid, quinic acid, cis-aconitic acid, itaconic acid, propionic acid, sebacic acid, succinamic acid, L-pyroglutamic acid, L-aspartic acid, L-glutamic acid, glycyl-L-aspartic acid, glycyl-L-glutamic acid, *p*-hydroxy phenylacetic acid, *γ*-hydroxybutyric acid, hydroxy-L-proline, L-leucine, L-alanyl-glycine, L-ornithine, L-phenylalanine, D-serine, D-galactonic acid lactone, D-alanine, L-threonine, D,L-carnitine, urocanic acid, *γ*-amino butyric acid, putrescine, uridine, phenyethylamine, 2-aminoethanol and 2,3-butanediol. The *mxaF* and *nifH* genes for, respectively, methanol dehydrogenase and nitrogenase reductase are present in the genomic DNA of the strains REICA_082^T^, REICA_032 and REICA_211. The genomic DNA G+C contents of strains REICA_082^T^ and REICA_032 are 52.9 and 52.7 mol%, respectively. The 16S rRNA and *rpoB* gene sequences were deposited under the accession numbers [GenBank:JF795011, JF795017] for REICA_082^T^, respectively.

The type strain, REICA_082^T^ (= LMG 26432 =NCCB 100390^T^), was isolated from internal root tissues of rice (*Oryza sativa* L.) cultivar APO. The roots were sampled at flowering stage from an experimental paddy field at the IRRI, Philippines.

## Methods

### Plant material and strain isolation

Rice (*Oryza sativa* L.) plants (cultivar APO) were sampled from a managed (rotary spading, once yearly) loamy paddy field, located at the International Rice Research Institute (IRRI), Los Baños, The Philippines. Replicate roots (150 g) devoid of rhizosphere soil were surface-sterilized and endophytic bacterial cell pellets obtained as described previously [29]. These replicate pellets were used for further isolation by plating, after maximally two days. Strains REICA_142^T^ (=LMG 26429^T^ =NCCB 100393^T^), REICA_084 (=LMG 26431 =NCCB 100392), REICA_191 (=LMG 26430 =NCCB 100394), REICA_082^T^ (=LMG 26432^T^ =NCCB 100390^T^), REICA_032 (=LMG 26433 =NCCB 100389) and REICA_211 (=LMG 26434 =NCCB 100391) were thus isolated, as independent (non-clonal) isolates based on their different origins, on R2A agar medium (BD – Difco, Detroit, USA), following incubation at 28°C for 3 days. All strains were then streaked to purity, after which cultures were stocked in 20% glycerol at −80°C.

### Phylogenetic analyses

All six strains were subjected to genomic DNA extraction using the Wizard genomic DNA purification kit (PROMEGA, Madison, WI, USA). Strains were presumptively identified by amplifying the 16S rRNA gene with the universal primers 8F and 1492R as described [30]. The resulting sequences were determined in an ABI 377 DNA sequencer (Applied Biosystems), after which they were assembled using DNA baser software (Heracle BioSoft). The identities of the almost-complete 16S rRNA gene sequences (minimum of 1,301 bp) were determined by alignment with sequences from the Silva database (*SSU Ref NR 111*, *July 2012*) [31] using the SINA aligner v1.2.9 (http://www.arb-silva.de/aligner/). Alignments were refined by visual inspection. All positions with ambiguously-aligned positions (i.e. adjacent columns without conserved positions) were removed. The evolutionary history of these sequences in the context of 41 closely related taxa were inferred using a Maximum Parsimony (MP) algorithm. Trees were calculated using the complete deletion option, all codon positions and a CNI level of 3 with an initial tree by random addition of sequences (100 replicates) from MEGA 5.0 software [32]. The robustness of the trees was assessed using 1000 bootstrap repetitions and a random seed. Clades were considered to have high nodal support if the associated taxa clustered together more than 50% in the bootstrap resampling tests. The confidence level of each node was determined by building a consensus tree of 100 maximum parsimony trees from bootstrap pseudoreplicates of the original data set.

Moreover, *rpoB* gene fragments were amplified from the set of six strains by targeting the highly variable region between positions 1300 and 2400 using primers CM_7_ and CM_31b_[16]. The resulting fragments were then sequenced using standard techniques. The partial *rpoB* gene sequences from the six novel strains were then compared to those from (1) 209 members of the *Enterobacteriaceae* retrieved from the Integrated Microbial Genomes (database v.3.2, http://img.jgi.doe.gov/cgi-bin/w/main.cgi), (2) 94 *Enterobacter*-related sequences [16,23] and (3) 18 publicly-available *Enterobacteriaceae* type strains. Sequences were compared at the DNA level, but were also translated to create a predicted amino acid sequence data set. Then, alignments were performed using ClustalW (MEGA v5.0; [32]). Alignment inspection and phylogenetic analyses were done as described above. Finally, a consensus tree was built on the basis of the alignments, using 45 closely-related taxa.

### DNA:DNA hybridization assays

To assess whether the six novel strains represent novel species within the genus *Enterobacter*, four strains, i.e. REICA_032, REICA_082^T^, REICA_142^T^ and REICA_191, were selected for comparison, by paired whole genome hybridizations, with the type strains of the closest defined *Enterobacter* species (based on the congruent results of the phylogenetic analyses), i.e. *E. radicincitans* LMG 23767^T^, *E. oryzae* LMG 24251^T^, *E. arachidis* LMG 26131^T^ and *E. cowanii* LMG 23569^T^ (University of Ghent, Laboratory for microbiology, Ghent, Belgium). Multiple well-isolated colonies from each strain were subjected to genomic DNA extraction [33]. Hybridizations were performed in the presence of 50% formamide at 45°C, according to a modification of the method described by Ezaki et al. [34], and fluorescence measurements used for detection. The DNA:DNA relatedness percentages reported are the means of at least four hybridizations. The values given between brackets are the differences between the reciprocal values.

### Characteristics used for further identification

The six strains, as compared to the type strains of the closest related species, were further morphologically, biochemically, chemotaxonomically and physiologically characterized according to standard methods as described by Gerhardt et al. [35]. Colony morphology was determined using trypticase soy agar (TSA; BD – Difco, Detroit, USA) as the growth medium. Cellular morphology and motility were examined by phase contrast microscopy (Carl Zeiss, Jena, Germany). Cell dimensions were measured with a 10× ocular and 100× objective (/1.25). Confirmatory motility tests were performed in R2A broth solidified with 0.4% agar in accordance with Gerhardt et al. [35]. Gram staining was carried out with a standard Gram staining kit (Sigma-Aldrich, Steinheim, Germany).

For cellular fatty acid analysis, the six novel strains, next to three type strains from species of the genus *Enterobacter* (i.e. *E. cloacae* subsp. *cloacae* ATCC 13047^T^, *E. radicincitans *D5/23^T^ and *E. arachidis* Ah-143^T^) were cultivated in triplicate on plates containing TSB (trypticase soy broth) amended with 15 g of agar (TSBA) at 30°C for about 24 h. Fatty acid methyl esters (FAME) from strains at the same physiological stage were extracted and prepared by the instant FAME^TM^ protocol of the Microbial Identification System (MSI, Microbial ID, Inc., Newark, Delaware, USA; http://www.midi-inc.com/pages/mis_literature.html). The extracts were analyzed by using Agilent 6890 (Agilent Technologies, USA) with a flame ionization detector after capillary column (Ultra 2, 25 m, 0.20 mm, 0.33 μm - phenyl methyl silicon fused silica, Agilent Technologies) separation. The rapid ITSA1 method for environmental samples was used. The samples (2 μL) were injected in split mode (1:20), with injection temperature of 250°C and carrier gas hydrogen. The temperature regime of the column was 170°C – 28°C min^-1^; 288°C − 60°C min^-1^ ; 310°C − 1.25 min (GC run time was 5.831 min). The FAME profiles were identified by MIS Sherlock software (ITSA1 Library v.1.1); unweighed pair-grouping based dendrograms were generated using Euclidian distance from the closest strains retrieved from Sherlock Library Generation Software.

The effects of different temperatures on growth were determined using R2A agar plates (Difco, Detroit, USA) incubated at 8, 15, 23, 28, 30, 37, 42, 50 and 65°C. Salt tolerance was tested in a concentration range of 1, 2.5, 5, 7.5 and 10% NaCl (w/v) in R2A broth incubated at 37°C. Tests for resistance to ampicillin, chloramphenicol, colistin sulphate, kanamycin, nalidixic acid, nitrofurantoin, streptomycin and tetracycline were performed using Mastring-S M26 antibiotic discs (Mast diagnostic, Bootle, UK), while resistances to rifampicin (25 ug ml^-1^) and gentamicin (25 ug ml^-1^) were evaluated separately. These tests were performed on both R2A and LB agar using inocula of 8 *log* cells Petri dish^-1^ (former tests) or varied cell numbers (latter tests) at 28°C. Strains were considered to be resistant when the inhibition zones around the disks were below 10 mm or when growth of single non-mutant colonies was detected (for rifampicin and gentamicin tests) after 48 h.

Basic biochemical characteristics such as arginine dehydrolase, ornithine and lysine decarboxylase were tested in Moeller’s broth using incubation for 96 h at 30°C as described [35]. Testing for oxidase activity was performed on the relevant test discs, for urease activity in urea broth and for production of hydrogen sulfide on sulfide test strips following the manufacturer’s instructions (Fluka, Buchs, Switzerland). Tests for indole production, esculin hydrolysis, citrate degradation (on Simmon’s agar) and gluconate dehydrogenase were performed at 30°C and read after 24 h, as described [35]. Malonate decarboxylase tests were read after 48 h at 30°C. Methyl red and Voges-Proskauer tests were read at after 48 h at 37°C [35]. Production of acetoin and 2-ketogluconate were inferred from the Voges-Proskauer and gluconate dehydrogenase activity tests, respectively.

In addition, strains REICA_142^T^ and REICA_082^T^ were subjected to biochemical identification using API-20E test strips (BioMérieux Inc., France). Strips were inoculated using a suspension prepared from a one-day-old well-isolated colony and the inoculated strips were incubated at 37°C for 24 h according to the manufacturer’s instructions. The results were converted into 7-digit numerical profiles and strains were identified using the analytical profile index (API) database v4.0 (http://www.biomerieux-usa.com). Furthermore, the broad utilization of carbonaceous compounds was determined using Biolog GN2 microplates (Hayward, USA) after an incubation period of 48 h at 28°C.

### Plant-growth-promoting (PGP) properties

Several PGP properties of the bacterial strains in relation to the host plant were investigated on the basis of pure culture studies. The production of indole-3-acetic acid (IAA) [36] and fixation of atmospheric N_2_[7] were evaluated by standard methods in test tubes after incubation at 30 and 37°C, respectively. The production of siderophores [37], amylases, cellulases and proteases, as well as the solubilization of phosphate [35,38] were tested on the respective prescribed media. Furthermore, growth tests on so-called “copiotrophic” and “oligotrophic” media [39], on DF (Dworking and Foster) salt with 1-aminocyclopropane-1-carboxylate (ACC) as the sole nitrogen source [40] and on modified M9 salt agar amended with 1% (v/v) methanol and 0.3% (w/v) NH_4_ as sole carbon and nitrogen sources [41] were performed using Petri dishes and 5 days of incubation at 37°C. Using genomic DNA templates, PCR-based tests for the presence of the *mxaF* and *nifH* genes, encoding, respectively, the large subunit of methanol dehydrogenase and nitrogenase reductase, were also performed. The two genes were thus amplified with primer pairs maxF-f1003 – maxF-r1561 [42] and PolF - PolR [43], using the PCR conditions described in both references.

Colonization of rice plants was evaluated *in vivo* using a rifampicin-resistant mutant of strain REICA_142^T^, denoted REICA_142^TR^. The mutant was selected on R2A agar medium amended with 25 μg ml^-1^ rifampicin (Sigma-Aldrich, St. Louis, MO) and streaked to purity. One-day-old germinated rice seeds were incubated for 1 h with 8.4 *log* cells of REICA_142^TR^ CFU ml^-1^ (REICA_142^TR^ treatment) or with sterile phosphate buffer solution (pH 6.5; control treatment) [44]. For each treatment, four replicate rice seedlings were grown in autoclaved as well as natural V soil [45] for up to 4 weeks at 70% water holding capacity. Water lost from the pots was replaced daily using sterile demineralized water. Following growth, all rice plants were surface-sterilized [46], rice tissue was treated with mortar and pestle, after which serial dilutions of the resulting homogenates were made and plated onto selective agar (R2A supplemented with Rif). Following plate incubations at 28°C for 72 h, the bacterial communities obtained from the plant tissue were enumerated. The ability of strain REICA_142^TR^ to invade rice plants from the V soil was thus confirmed by isolating colonies from the relevant plates (at least one per replicate) and performing BOX-A1R PCR on these [47].

#### Availability of supporting data

The accession numbers for the 16S rRNA gene sequences of *Enterobacter oryziphilus* strains REICA_084, REICA_142^T^ and REICA_191 are [GenBank:JF795012, JF795013, JF795014], and of *Enterobacter oryzendophyticus* strains REICA_032, REICA_082^T^ and REICA_211 are [GenBank:JF795010, JF795011, JF795015], respectively. The accession numbers for the *rpoB* gene sequences of strains REICA_084, REICA_142^T^ and REICA_191 are JF795018, JF795019 and JF795020, and of *Enterobacter oryzendophyticus* strains REICA_032, REICA_082^T^ and REICA_211 are JF795016, JF795017 and JF795021, respectively.

The generated phylogenetic trees from the 16S rRNA and rpoB genes were deposited in the publicly-accessible TreeBASE data repository with the project number 14166.

## Competing interests

The authors declare that they have no competing interests.

## Authors’ contributions

PRH was the major contributor to the whole study, being active from the design of the experiments to the final revision of the manuscript. RN carried out some of the taxonomic analyses. DE performed the FAME analysis. EK constructed the phylogenetic trees and helped in the final version of the manuscript. AS, LSvanO and JDvanE designed the sampling strategy, collaborated in the data analyses and revised the manuscript. All authors read and approved the final manuscript.

## Supplementary Material

Additional file 2: Figure S2 Maximum-likelihood tree based on *rpoB* gene sequences showing the phylogenetic position of *Enterobacter oryziphilus* sp. nov. and *Enterobacter oryzendophyticus* sp. nov. within the genus *Enterobacter*. A total of 45 nucleotide sequences (with 56 variable positions from a total of 495) were used, scoring the arithmetic means of log likelihood -3536.24. The nodes in terminal branches supported by ≥ 50% of the ML bootstrap analysis and homogeneous Bayesian (BI) posterior probabilities are shown. The tree is drawn to scale with bar indicating 0.06% substitutions per nucleotide position. Sequences from *Pantoea* genus were used as outgroup. Click here for file

Additional file 3: Table S1 Fatty acid profiles of strains REICA_142^T^, REICA_084, REICA_191, REICA_082^T^, REICA_032, REICA_211 and type strains of closely related species of the genus *Enterobacter* measured by gas chromatography. Click here for file

Additional file 1: Figure S1 Maximum-likelihood tree based on nearly complete 16S rRNA gene sequences showing the phylogenetic position of *Enterobacter oryziphilus* sp. nov. and *Enterobacter oryzendophyticus* sp. nov. within the genus *Enterobacter*. A total of 41 nucleotide sequences (with 131 variable positions from a total of 1125) were used, scoring the arithmetic means of log likelihood -3228. The nodes in terminal branches supported by ≥ 50% of the ML bootstrap analysis and homogeneous Bayesian (BI) posterior probabilities are shown. The tree is drawn to scale with bar indicating 0.05% substitutions per nucleotide position. Sequences from *Pantoea* genus were used as outgroup. Click here for file

Additional file 4: Figure S3 Dendrogram derived from the fatty acid (FA) patterns showing the positions of *Enterobacter oryziphilus* sp. nov. and *Enterobacter oryzendophyticus* sp. nov. within the *Enterobacteriaceae*. Click here for file
