# Supplementary figures and images for: The new species Enterobacter oryziphilus sp. nov. and Enterobacter oryzendophyticus sp. nov. are key inhabitants of the endosphere of rice
Source: BMC Microbiol. 2013 Jul 16;13:164. doi: 10.1186/1471-2180-13-164 (PMC3728145; doi:10.1186/1471-2180-13-164)

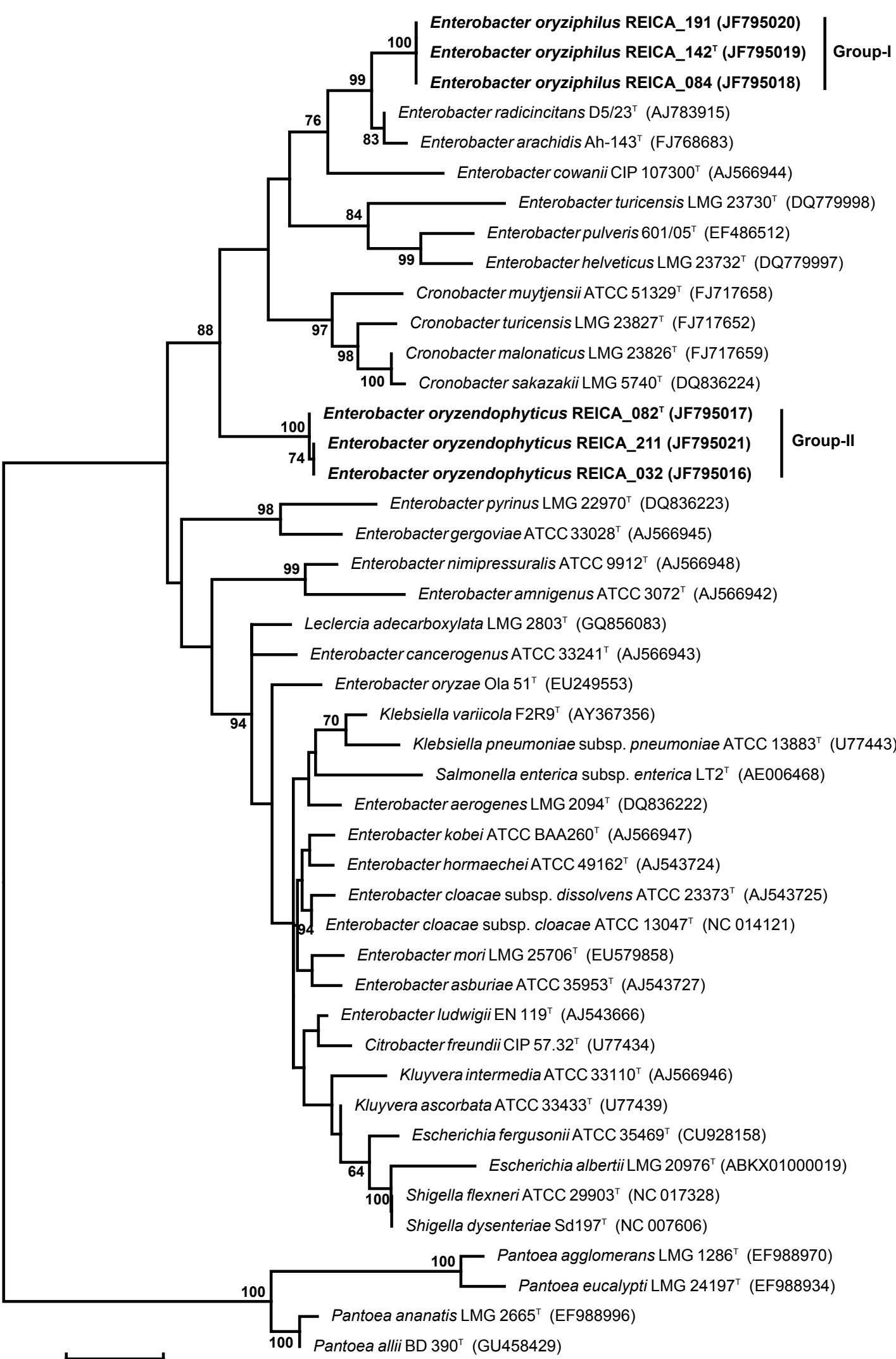

0.05

Supplement: Additional file 2: Figure S2 — Maximum-likelihood tree based on rpoB gene sequences showing the phylogenetic position of Enterobacter oryziphilus sp. nov. and Enterobacter oryzendophyticus sp. nov. within the genus Enterobacter. A total of 45 nucleotide sequences (with 56 variable positions from a total of 495) were used, scoring the arithmetic means of log likelihood -3536.24. The nodes in terminal branches supported by ≥ 50% of the ML bootstrap analysis and homogeneous Bayesian (BI) posterior probabilities are shown. The tree is drawn to scale with bar indicating 0.06% substitutions per nucleotide position. Sequences from Pantoea genus were used as outgroup. [file 1471-2180-13-164-S2.pdf]

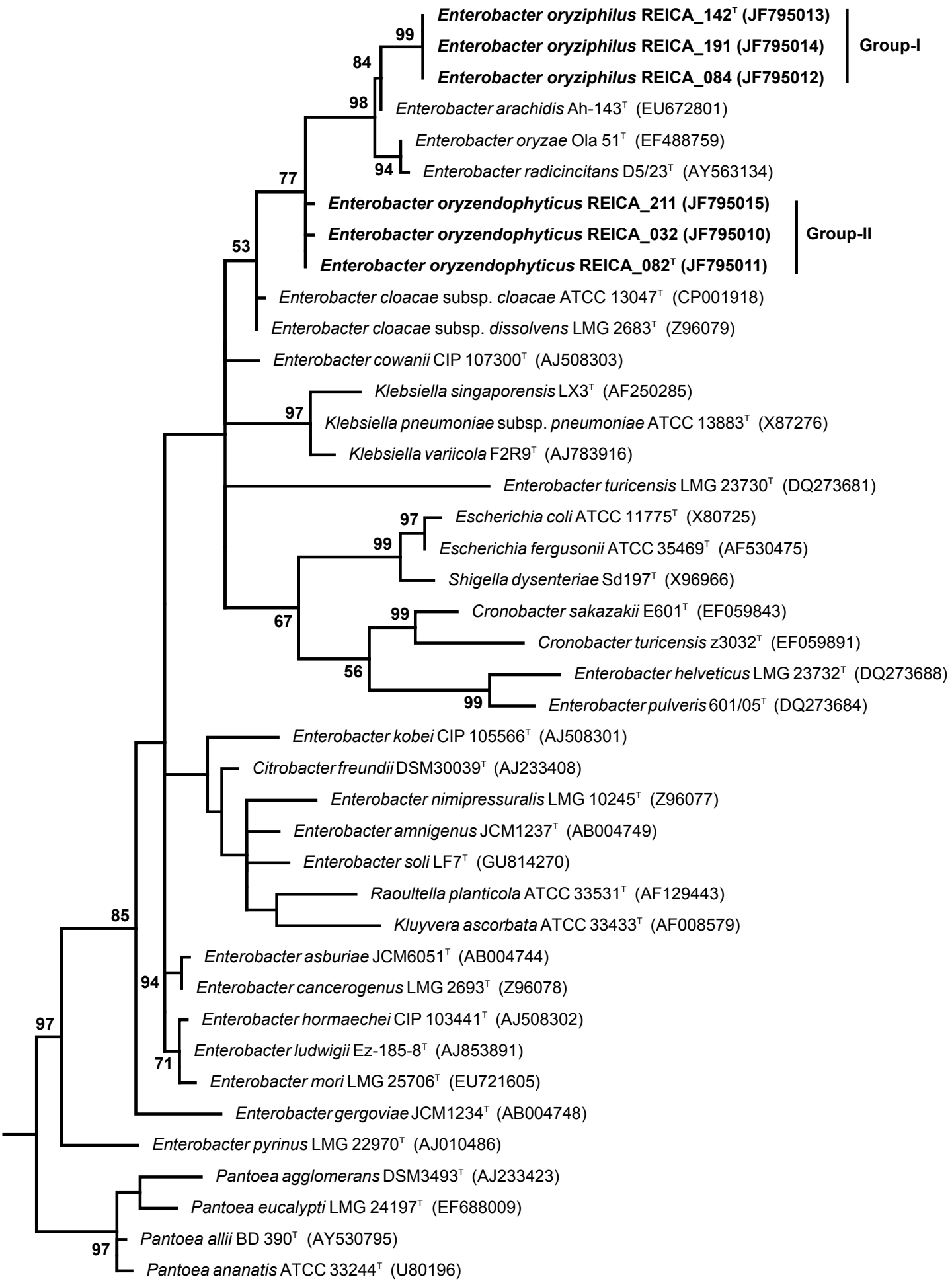

0.005

Supplement: Additional file 1: Figure S1 — Maximum-likelihood tree based on nearly complete 16S rRNA gene sequences showing the phylogenetic position of Enterobacter oryziphilus sp. nov. and Enterobacter oryzendophyticus sp. nov. within the genus Enterobacter. A total of 41 nucleotide sequences (with 131 variable positions from a total of 1125) were used, scoring the arithmetic means of log likelihood -3228. The nodes in terminal branches supported by ≥ 50% of the ML bootstrap analysis and homogeneous Bayesian (BI) posterior probabilities are shown. The tree is drawn to scale with bar indicating 0.05% substitutions per nucleotide position. Sequences from Pantoea genus were used as outgroup. [file 1471-2180-13-164-S1.pdf]

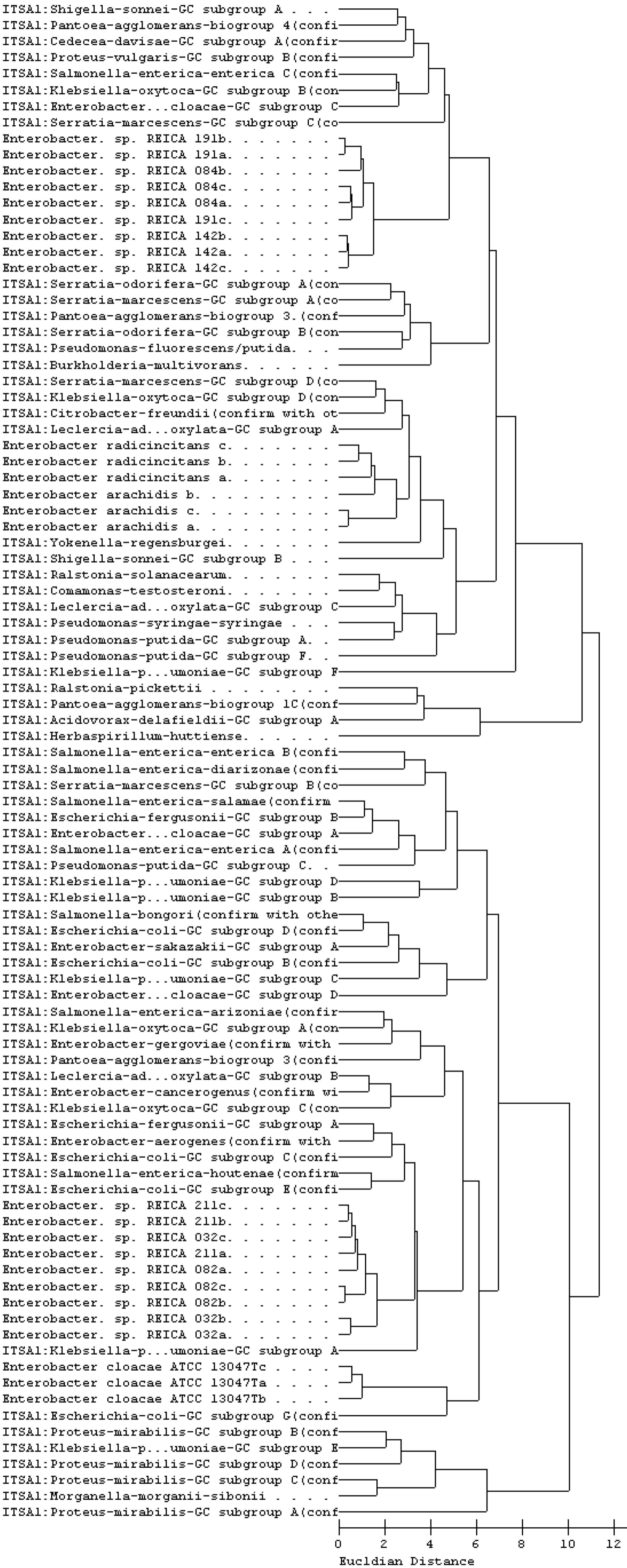

Supplement: Additional file 4: Figure S3 — Dendrogram derived from the fatty acid (FA) patterns showing the positions of Enterobacter oryziphilus sp. nov. and Enterobacter oryzendophyticus sp. nov. within the Enterobacteriaceae. [file 1471-2180-13-164-S4.pdf]
