# Supplementary material for: The new species Enterobacter oryziphilus sp. nov. and Enterobacter oryzendophyticus sp. nov. are key inhabitants of the endosphere of rice
Source: BMC Microbiol. 2013 Jul 16;13:164. doi: 10.1186/1471-2180-13-164 (PMC3728145; doi:10.1186/1471-2180-13-164)
Supplement: Additional file 3: Table S1 — Fatty acid profiles of strains REICA_142T, REICA_084, REICA_191, REICA_082T, REICA_032, REICA_211 and type strains of closely related species of the genus Enterobacter measured by gas chromatography. [file 1471-2180-13-164-S3.docx]

**Supplementary Table S1**. Fatty acid profiles of strains REICA_142^T^, REICA_084, REICA_191, REICA_082^T^, REICA_032, REICA_211 and type strains of closely related species of the genus *Enterobacter* measured by gas chromatography. Three single well-isolated colonies were used as replicates

Taxa: 1, REICA_142^T^; 2, REICA_084; 3, REICA_191; 4, REICA_082^T^; 5, REICA_032; 6, REICA_211; 7, *E. cloacae* ATCC 13047^T^; 8, *E. radicincitans* D5/23^T^; 9, *E. arachidis* Ah-143^T^

| **Average** | **1** | **2** | **3** | **4** | **5** | **6** | **7** | **8** | **9** |
| --- | --- | --- | --- | --- | --- | --- | --- | --- | --- |
| **Saturated** |  |  |  |  |  |  |  |  |  |
| 10:0 | 0.00 | 0.03 | 0.03 | 0.00 | 0.00 | 0.00 | 0.00 | 0.00 | 1.08 |
| 12:0 | 2.15 | 2.25 | 2.12 | 1.62 | 1.99 | 1.74 | 1.00 | 1.68 | 2.53 |
| 13:0 | 0.04 | 0.03 | 0.04 | 0.06 | 0.17 | 0.05 | 0.52 | 0.15 | 1.42 |
| 14:0 | 3.98 | 4.13 | 4.16 | 2.76 | 2.80 | 2.77 | 3.42 | 2.83 | 3.11 |
| 15:0 | 0.89 | 0.62 | 0.85 | 0.55 | 0.38 | 0.56 | 6.81 | 3.15 | 2.30 |
| 16:0 | 34.28 | 34.89 | 34.89 | 32.65 | 32.86 | 33.33 | 28.64 | 20.53 | 22.08 |
| 17:0 | 0.47 | 0.29 | 0.39 | 0.32 | 0.35 | 0.46 | 3.00 | 0.91 | 2.35 |
| 18:0 | 0.32 | 0.32 | 0.29 | 0.40 | 0.44 | 0.45 | 0.39 | 0.39 | 2.10 |
| **Unsaturated** |  |  |  |  |  |  |  |  |  |
| 15:1 ω8c | 0.00 | 0.05 | 0.00 | 0.04 | 0.00 | 0.00 | 0.23 | 0.12 | 1.58 |
| 15:1 ω6c | 0.00 | 0.00 | 0.00 | 0.00 | 0.43 | 0.00 | 0.08 | 0.05 | 0.00 |
| 15:1 ω5c | 0.09 | 0.07 | 0.06 | 0.06 | 0.04 | 0.10 | 0.01 | 0.11 | 1.64 |
| 16:1 ω7c/16:1 ω6c | 20.68 | 24.55 | 22.52 | 26.38 | 24.63 | 23.64 | 15.02 | 11.28 | 14.95 |
| 16:1ω5c | 0.14 | 0.14 | 0.14 | 0.25 | 0.24 | 0.23 | 0.29 | 13.25 | 1.86 |
| 17:1 ω8c | 0.13 | 0.09 | 0.10 | 0.06 | 0.16 | 0.08 | 0.35 | 6.50 | 1.91 |
| 18:1 ω7c/18:1ω6c | 19.24 | 18.82 | 18.28 | 27.56 | 26.91 | 26.50 | 21.07 | 21.15 | 15.49 |
| 18:1 ω5c | 0.10 | 0.10 | 0.09 | 0.10 | 0.10 | 0.09 | 0.00 | 0.02 | 0.03 |
| 20:1 ω7c | 0.06 | 0.05 | 0.05 | 0.04 | 0.02 | 0.05 | 0.03 | 0.00 | 2.14 |
| **Hydroxylated** |  |  |  |  |  |  |  |  |  |
| 15:0 3OH | 0.07 | 0.09 | 0.02 | 0.02 | 0.20 | 0.00 | 0.16 | 0.15 | 1.80 |
| 14:0 3OH/16:1 iso I | 0.81 | 0.81 | 0.75 | 0.85 | 0.43 | 0.92 | 1.21 | 1.25 | 2.55 |
| **Cyclopropyl-Branched** |  |  |  |  |  |  |  |  |  |
| 17:0 cyclo | 14.20 | 10.27 | 12.93 | 4.85 | 5.93 | 7.30 | 15.55 | 14.46 | 13.42 |
| 19:0 cyclo | 0.62 | 0.55 | 0.55 | 0.22 | 0.22 | 0.27 | 1.49 | 1.08 | 3.11 |
| **unknown FA (ECL 14.502)** | 1.72 | 1.88 | 1.71 | 1.22 | 1.71 | 1.44 | 0.72 | 0.95 | 2.57 |
| **Standard deviation** | *1* | *2* | *3* | *4* | *5* | *6* | *8* | *9* | *7* |
| ***Saturated*** |  |  |  |  |  |  |  |  |  |
| *10:0* | *0.00* | *0.02* | *0.03* | *0.00* | *0.00* | *0.95* | *0.00* | *0.00* | *1.83* |
| *12:0* | *0.03* | *0.10* | *0.04* | *0.02* | *0.10* | *0.90* | *0.03* | *0.29* | *1.13* |
| *13:0* | *0.00* | *0.02* | *0.01* | *0.02* | *0.29* | *1.55* | *0.02* | *0.04* | *2.36* |
| *14:0* | *0.06* | *0.17* | *0.10* | *0.06* | *0.04* | *1.58* | *0.02* | *0.43* | *1.18* |
| *15:0* | *0.19* | *0.21* | *0.12* | *0.12* | *0.16* | *0.03* | *0.13* | *0.55* | *2.17* |
| *16:0* | *0.35* | *0.41* | *0.50* | *0.14* | *0.11* | *0.23* | *0.06* | *17.79* | *14.71* |
| *17:0* | *0.04* | *0.11* | *0.09* | *0.07* | *0.16* | *0.03* | *0.27* | *0.84* | *2.67* |
| *18:0* | *0.01* | *0.01* | *0.01* | *0.01* | *0.01* | *0.01* | *0.01* | *0.07* | *3.15* |
| ***Unsaturated*** |  |  |  |  |  |  |  |  |  |
| *15:1 ω8c* | *0.00* | *0.08* | *0.00* | *0.06* | *0.00* | *0.00* | *0.01* | *0.01* | *2.72* |
| *15:1 ω6c* | *0.00* | *0.00* | *0.00* | *0.00* | *0.75* | *0.00* | *0.01* | *0.00* | *0.00* |
| *15:1 ω5c* | *0.04* | *0.01* | *0.01* | *0.01* | *0.04* | *0.00* | *0.02* | *0.03* | *2.71* |
| ***16:1 ω7c/16:1 ω6c*** | *1.28* | *1.96* | *2.24* | *1.09* | *1.04* | *0.16* | *0.12* | *9.55* | *9.27* |
| *16:1ω5c* | *0.02* | *0.00* | *0.01* | *0.00* | *0.01* | *0.01* | *0.01* | *22.55* | *2.79* |
| *17:1 ω8c* | *0.02* | *0.01* | *0.02* | *0.01* | *0.19* | *0.01* | *0.05* | *10.67* | *2.99* |
| *18:1 ω7c* | *0.22* | *0.19* | *0.05* | *0.55* | *0.21* | *0.28* | *0.23* | *3.07* | *8.50* |
| *18:1 ω5c* | *0.00* | *0.01* | *0.00* | *0.00* | *0.01* | *0.00* | *0.00* | *0.04* | *0.04* |
| *20:1 ω7c* | *0.00* | *0.01* | *0.01* | *0.00* | *0.03* | *0.03* | *0.03* | *0.00* | *3.64* |
| ***Hydroxylated*** |  |  |  |  |  |  |  |  |  |
| *15:0 3OH* | *0.08* | *0.06* | *0.02* | *0.02* | *0.06* | *0.00* | *0.06* | *0.13* | *3.01* |
| *14:0 3OH/16:1 iso I* | *0.71* | *0.21* | *0.09* | *0.04* | *0.25* | *0.08* | *0.36* | *0.14* | *2.09* |
| ***Cyclopropyl-Branched*** |  |  |  |  |  |  |  |  |  |
| *17:0 cyclo* | *1.08* | *2.12* | *2.03* | *1.14* | *1.27* | *0.31* | *0.40* | *11.58* | *7.71* |
| *19:0 cyclo* | *0.50* | *0.20* | *0.52* | *0.06* | *0.04* | *0.03* | *0.11* | *0.10* | *2.65* |
| ***unknown FA (ECL 14.502)*** | *0.03* | *0.06* | *0.05* | *0.05* | *0.24* | *0.21* | *0.06* | *0.15* | *1.78* |

The new species *Enterobacter oryziphilus* sp. nov. and *Enterobacter oryzendophyticus* sp. nov. are key inhabitants of the endosphere of rice^.^ **BMC Microbiology.** Pablo Rodrigo Hardoim, Rashid Nazir, Angela Sessitsch, Dana Elhottová, Elisa Korenblum, Leonard Simon van Overbeek, Jan Dirk van Elsas. **Corresponding author**: Pablo R. Hardoim, *Centre of Marine Science, University of Algarve, 8005-139, Faro, Portugal*; E-mail: [phardoim@gmail.com](mailto:phardoim@gmail.com)
